# Developing a methodological tool for exploring sense of safety in religious spaces

**DOI:** 10.3389/fpsyg.2025.1448951

**Published:** 2025-04-22

**Authors:** Anne Birgitta Pessi, Henrietta Grönlund, Ruth Illman, Ritva Palmén, Meri-Anna Paloniemi, Teemu Pauha, Jenni Spännäri

**Affiliations:** ^1^Church and Social Studies, Department of Practical Theology, Faculty of Theology, University of Helsinki, Helsinki, Finland; ^2^The Donner Institute for Research Into Religion and Culture, Turku, Finland

**Keywords:** religion, space, embodiment, emotions, sense of safety, methods

## Abstract

Emotions are a fundamental part of human existence, a power that massively affects our thinking and actions. Even after the affective turn in social sciences, religion is to a very large extent overlooked in the sociology of emotions. Then, psychological research on sense of safety often leaves the societal and political contexts of emotions unattended. Sense of safety—the topic of our study—provides an excellent topic to explore emotions as social, societal, spatial, and embodied phenomenon. Our article concerns the ways in which sense of safety is both constructed and contested in religious spaces and how to study the topic. The aim of this article thus is to develop a methodological tool for empirically exploring the sense of safety experienced in the spaces of religion. The article first discusses sense of safety and space, specifically in relation to religion, and the need for a methodological approach to investigating it empirically. The article leans on environmental psychology, urban studies, and research on the recognition and politics of belonging from political philosophy. Based on this, we design The Spiral Model: a one-plus-five dimensions tool for empirical exploration of sense of safety in religious spaces, and the dimensions are: Identifying a religious place; Unpacking intergroup connectedness, and networks of belonging and safety; Focusing on intragroup boundaries, and how they are afforded by physical surroundings; Exploring the embodied emotions that are associated with the place and its spatial dimension; and, Looking at the embodied emotions of sense of safety of inter- and intragroup nexuses in the framework of wider social, societal, and global vistas. To demonstrate how the model can be applied, for both data collection and analysis, we introduce four ongoing, collaborative empirical case studies: (1) a novel communal church building, (2) LGBTQ+ Muslims, (3) Jewish mikveh baths, and (4) intersections of dance and religion. Although the spiral model developed in this article is far from complete, it holds a lot of potential for advancing a more holistic view of humans in research and deepening the understanding of social space with philosophical conceptualization and analysis related to recognition and politics of belonging.

## 1 Introduction

This article concerns the ways in which sense of safety is both constructed and contested in religious spaces and how to study the topic. Sense of safety as all emotions are a fundamental part of human existence, a power that massively affects our thinking and actions (Ekman and Davidson, 1994). Emotions are biologically based psychological states (Damasio, 1998; Cabanac, 2002), and constructed brain–body phenomena (Feldman Barrett, 2017), yet never only about individuals. Emotions are experienced individually but influenced by social factors near and far: by the emotions of our close ones, by social norms, by cultural values, et cetera. They are “constructions of the world, not reactions to it,” as Feldman Barrett (2017).

We live in the midst of emotional regimes, all through human societies (Nussbaum, 2001; Riis and Woodhead, 2010, pp. 10, 47–51; Wahl-Jorgensen, 2019, pp. 9, 115). Furthermore, emotions take place in spaces. Humans are embodied creatures living in spatial realities, and emotions must be examined as embodied practices (McGuire, 2008; Vásquez, 2011). Emotions condense in spaces, and spaces make them tangible (Dilger et al., 2020; Finlayson, 2012; Burchardt and Westendorp, 2018; Bradamat et al., 2022; Knott et al., 2016).

In this study, we recognize the variety in the ways emotions and its neighbouring concepts such as feelings, affect or mood are examined in research (e.g., Moors, 2022). We approach emotions as complex reaction patterns, involving experiential, behavioral, and physiological elements, contrasted with our understanding of feelings as self-contained phenomenal experiences. Where we view feelings as essentially mental, emotions have the quality of engaging with the world (see also APA Dictionary of Psychology).

Sense of safety provides an excellent topic to explore emotions as a social, societal, and spatial phenomenon. Albeit being experienced by individuals, sense of safety is deeply embodied, inter-human, and centers around both belonging and non-belonging. It is an individual’s internal feeling about safety, accompanied by a subjective perception of objective events (Zou and Meng, 2020; Nilsen et al., 2004). Sense of safety centers around a feeling of being threatened or not (Suojanen, 2022, p. 35), and takes place under particular spatial–temporal conditions (e.g., Collins and Guidry, 2018). According to Johanna Lynch (2020), sense of safety is a holistic state of being in which a variety of basic human needs and their fulfillment are integrated; sense of safety is about feeling bodily calm, being in a safe surrounding, feeling valued and valuable, and being allowed to express one’s full potential. As sense of safety is about the general state of the whole organism, it can be threatened at either bodily, social, or psychological level. According to Lynch (2020), sense of safety can be undermined by impending bodily harm, social shame, or loss of hope and meaning. On the contrary, the experiences of love, recognition, comfort, and courage within our social relationships lay a vital foundation for our self-realization and self-esteem. These experiences enable us to cultivate our practical identities and view ourselves as interacting, moral individuals with unique traits and distinct roles in society, each possessing our own beliefs, aspirations, needs, and goals (Zurn, 2015; Lynch, 2020). In research, few concrete methodological tools exist to empirically explore the sense of safety as an embodied, spatial phenomenon. Here lies our interest.

For the explorations of sense of safety, one particularly multifaceted context exists, fascinating both societally and psychologically: religious spaces, both material and mental. This duality most definitely concerns not only religious institutions themselves but societies at large. For instance, many recent surveys conducted by the European Union (FRA, 2023) show that antisemitism is widespread in Europe, as well as elsewhere in the world (AWR, 2022), escalating drastically during the fall of 2023 due to the violent conflict in Israel (e.g., Graham and Boyd, 2023). Antisemitism can be described as both a persisting and a constantly changing latent structure of beliefs, sentiments, imagery, and actions manifest on structural, cultural, and individual levels in society (Fein, 1987, p. 67; Illman and Vuola, 2024). Currently, many Jews report personal experiences—negative comments, mistrust, threats, discrimination, or outright violence—targeted against them because they are Jewish.

Similarly, a recent report by the European Union Agency for Fundamental Rights demonstrates that racism towards Muslims is exceedingly common in Europe (FRA, 2024). According to the report, one-fourth of Muslims in the EU feel they have been discriminated against because of their religion in the past year, and more than one in five have experienced harassment. Discrimination, harassment, and outright violence are especially common against Muslim women who are ‘visibly Muslim’ by wearing religious clothing.

Each religion and religious space may serve as a particular context that elicits a complex array of emotions, ranging from feelings of safety and belonging to struggles for power and underlying insecurities. Sense of safety comes very much from belonging (Lynch, 2020); Lynch et al., 2025. Belonging as a dynamic, emergent construct (Allen et al., 2021) essentially has both social and emotional (see, e.g., Lynch, 2020, p. 74) as well as embodied and spatial (see, e.g., Yuval-Davis, 2016, p. 199; Lynch, 2020, p. 93) dimensions. Negotiations of who belongs and who does not take place on all levels and contexts of society. These negotiations happen regarding competencies for belonging, opportunities to belong, motivations to belong and perceptions of belonging (see Allen et al., 2021).

Yet, religions are particularly contested and sensitive arenas of emotions and belonging/shutting out (see, e.g., Skidmore et al., 2023). Even mere religious buildings are never just buildings but symbols of different viewpoints, experiences, and discourses. The core theme of this article is the ways in which sense of safety is both constructed and contested in religious spaces and how to study the topic. Not enough academic tools exist. Even after the affective turn in social sciences and particularly in sociology (e.g., Riis and Woodhead, 2010), religion is to a very large extent overlooked in the sociology of emotions. On the other hand, psychological research on sense of safety often leaves the societal and political contexts of emotions unattended. Furthermore, in the body of research related to space and religion, emotions have not been a particular focus. Our approach combining emotions and space in religious contexts brings together the physical/embodied, social, and mental dimensions of space—and of emotions.

The aim of this article thus is: *to develop a methodological tool for empirically exploring the sense of safety experienced in the spaces of religion*—*a tool for both data collection and analysis*. We will carry out this aim via four steps: *(1) We first discuss sense of safety and space, specifically in relation to religion,* and the need for a methodological approach for investigating their interconnectedness empirically.

*Next, (2) we introduce methodological and conceptual approaches from three*—*one could say currently rising*—*fields, which provide viewpoints on the issue of space and sense of safety that can further our attempt to develop the methodological approach*. These are environmental psychology, urban studies, and research on recognition and politics of belonging from political philosophy. All in all, these three fields will inform and inspire us on how to develop the methodological tools to investigate sense of safety in religious spaces. Such bridging will also in itself enhance interdisciplinarity of spatial research of religion and sense of safety. Both environmental psychology and urban studies are often applied to promote design of spaces promoting wellbeing, including sense of safety. They view space as physical and socially constructed and examine the interplay between space, individual experiences, identities and belonging. Environmental psychology aims to understand how physical environments affect the human psyche and intertwine with identities and boundaries of belonging. Similarly, the viewpoints of spaces and the physical, material realities are central in urban studies, and often researched from the viewpoint of emotions and experiences related to them. We include the concepts of recognition and politics of belonging drawing from the viewpoint of political philosophy to further deepen our analytical approach to belonging. The exact reason for this is threefold and is explicated in the respective section below.

Then, the third and fourth steps in delivering our research aim: *(3) By building on this multifaceted constellation we will then proceed to introduce a preliminary spiral model of investigating sense of safety in religious spaces: a one-plus-five dimensions tool for empirical exploration of sense of safety in religious spaces.*

Following this, *(4) we will demonstrate how the model can be applied using four different empirical cases.* As this is a methodological article, we will not introduce empirical material, but descriptive cases where the model is utilized. Together these case studies hopefully illustrate the versatile opportunities of the introduced model. While we utilize research literature from different religions and different parts of the world, our case studies represent only certain religions and contexts in the global north. We absolutely recognize that the spiral model may not be applicable in relation to all religions and all contexts.

Understanding emotions and religion as intertwined, and their entanglements with the spatial, is much called for (e.g., Riis and Woodhead, 2010; Arweck, 2013; Maiese, 2011). The need for further methodological approaches and tools is paramount. Importantly, this does not only concern emotions in general, but also—and actually, in particular—sense of safety; Religions have a mammoth of power to generate both insecurity and sense of safety. Religions can be symbols of safety, belonging, and shared identities, as well as—and often simultaneously—of insecurity, exclusion, and differences. Such duality concerns both religious communities themselves and their relation to wider public space and religious diversity. In all cases, these emotions are about, or related to, exactly the sense of safety.

## 2 Explorations of sense of safety in relation to space—the case of religious spaces

The concept of space is multidimensional and contested. Most definitions however acknowledge the interplay between the material, social, and mental elements of space (e.g., Knott, 2014; who has developed spatial methodologies in relation to research on religion building on Lefebvre, among others). In this article, we use the concept of religious space to refer to buildings, sites, or geographical areas, which intertwine with religious social networks, meanings, and questions of power. In addition, we utilize the concept of place, which is often connected to subjective, experienced meanings. Thus, with religious places, we mean material, social, and mental spaces, which are religiously meaningful to a specific individual or group. A religious site or location can thus be defined as a space and/or a place, if it in the latter case has a specific religious meaning for an individual or group.

It is important to understand the concept of ‘space’ and spatiality as socio-physical phenomena. Thus, we view the embodied nature of emotions to concern not just tangible, physical spaces but spaces as socially constructed between individuals and groups (Durt et al., 2017). Knott (2009, 2010, 2014) has pioneered in developing a spatial approach in the study of religion, drawing from Lefebvre, among others. She emphasizes that any space is the sum of its material characteristics, the people who live in it and move through it, and the different representations and discourses associated with it. Spaces draw together physical/embodied, social, and mental dimensions (such as emotions), which we can analyze as we investigate specific places. Spaces also include traces of earlier times, different stratifications, and questions of power, struggles in which groups or individuals seek to express themselves (Knott, 2014). The physical, social, and mental dimensions of spaces need to be acknowledged when investigating how space is perceived, conceived, and lived. Thus, the spatial approach is not a method of data collection but a series of analytical steps.

All spaces compact some kind of emotions. Spaces are lived and their meanings negotiated, and these negotiations intertwine with embodied experiences and emotions, both in virtual and non-virtual contexts. As pointed out in the Introduction, emotions condense in spaces, and spaces make them tangible. There exists a body of research on religion and spatiality. Religious embodied social spaces combine layers of emotional regimes and make the contestations related to them tangible (Riis and Woodhead, 2010). For example, analyzing American Muslim women’s experiences of mosques, Mohammed (2024) demonstrated that over a third of them felt dissatisfied with the aesthetics, functionality, and maintenance of the mosque spaces allocated for women. This also affected their sense of safety; Mohammed’s informants, for instance, felt afraid to use the women’s entrance, which was typically located on the (often secluded and dark) backside of the building. While Mohammed’s (2024) findings illustrate how a religious space may be experienced differently by different (e.g., gendered) bodies, the research of spatial religion and geography of religion have to some extent ignored embodiment (Holloway, 2006; Knott et al., 2016).

Religions and religious spaces are a particularly powerful source and arena of emotions. The two-way street is apparent here: Identifications with religious traditions and communities include emotional aspects (e.g., sense of belonging, emotional commitment or rejection), and the social and societal dimensions of religions influence emotions and how they are interpreted and expressed (e.g., Dilger et al., 2020). Religions are truly a treasure box for researchers of emotions of several academic disciplines.

Interestingly, even if the ‘affective turn’ in sociology took place already three decades ago, religion is to a very large extent overlooked in the sociology of emotions. Then, on the other side, in the sociology of religion, emotions have been explored very modestly, even much less than in general sociology (Riis and Woodhead, 2010; Fer, 2018; Turner and Stets, 2005; Illman, 2018). For instance, there is research on the power of religion to divide and unite people but the exploration of the role of emotions has been more of a shadow zone. Then, in theological studies, vast majority of research on emotions takes place in systematic theology, not via empirical explorations. Empirical research, and tools for it, are needed.

Within the study of religion, emotions are also investigated in the context of the cognitive study of religion (CSR), particularly in relation to ritual (see, e.g., White et al., 2024; Whitehouse, 1996). Especially promising for future research are the so-called 4E models that conceptualize the human mind as ‘embodied, embedded, enactive, and extended,’ thus challenging cognitive psychology’s traditional view of the mind as enclosed in the brain (on 4E, see De Bruin et al., 2018). As suggested by the abbreviation, 4E models conceive of the mind as, on one hand, situated in the whole body and, on the other hand, extended into one’s environment. Accounting for such complexity in empirical research is challenging, however, and CSR studies into spatial religion have been few (but see Harmon-Vukić and Spitalnic, 2024).

Positively, however, particularly within ethnographic research on religion—also in virtual spaces, emotional aspects are increasingly highlighted in studies based on the analytical frameworks of lived or vernacular religion (see, e.g., Ammerman, 2021; Hintsala, 2016; Utriainen, 2020; Valk and Bowman, 2022; Winchester and Pagis, 2022). Illman (2018, pp. 4–9) has elucidated this trajectory by describing three theoretically relevant processes of change in ethnographic narratives: the ‘reflexive turn’ emphasising personal choice and self-realization; the ‘turn within’ highlighting emotions and embodiment; and the ‘turn to tradition’ that prompts an innovative search for inspiration and influences from history. These lines of development may seem contradictory as the first two are directed inward toward individual meanings and practices, the last outward toward community and tradition. However, these aspirations can be seen to be held together by a discourse of authenticity and aims to renew both traditions and personal engagements (Muir et al., 2023: 580–581), to balance in the crossroads. Hence, all the three turns can be traced as significant, intertwined narrative trajectories. Such re-orientation in the study of religions suggests a complex, nonbinary methodology where intellectual and emotional, cognitive, and embodied dimensions are integrated into a reflexive and comprehensive engagement (Illman, 2022, p. 137).

Also, the secular has been approached as spatial, material, and embodied, and in relation to emotions. For example, Scheer et al. (2019) suggest that the emotional containment expected from those working in public office can be viewed as a certain style of affectivity. It illustrates how the idea of ‘secular public’ is performed and can be coded as ‘neutral’ or ‘rational’ in opposition to the ‘irrational’ or ‘emotional’. They also argue that the repeated controversies over religion in public spaces mentioned above can be viewed as ‘secular affect’.

These entanglements are delicate and not always easily depicted, as many recent studies show, for instance, of religious minorities (Petersen, 2022; Wu et al., 2021), of religious communities facing crises like the Covid-19 pandemic (Edelman et al., 2021; Kolata, 2023; Schuerhoff, 2023; Vähäkangas, 2023), and of social exclusion (Alemanji et al., 2022; Schmidt, 2022). A pertinent example is Cutler (2006), who in their research on young-adult Jews show how a sense of safety, and threats to it, are negotiated and lived in minority–majority contexts even when opposition is not direct. These experiences relate to the historical persecution of Jews and experiences of ignorance about Judaism and Jewish life. The participants of the study used different emotion-work strategies to sustain a sense of safety, such as compartmentalizing their Jewish identities, distancing themselves emotionally from others, and humor. Exploring such phenomena, methodological sensitivity is essential.

## 3 Explorations of sense of safety in relation to space: environmental psychology

In contemporary psychology and social psychology, much emphasis is placed on studying the human mind in context. However, the context is very often limited to the immediate social surroundings and salient cultural traditions (Wapner and Demick, 2002, p. 3). In contrast, physical surroundings as well as built environments and spaces have been largely neglected. Writing in the context of social psychology, Meagher (2020, p. 3), for example, notes: ‘Recent trends in social psychology point to increased interest in extending current theories by better incorporating the body (e.g., embodied cognition) and the broader interpersonal context (e.g., situations). However, despite being a critical component in early social theorizing, the physical environment remains in large part underdeveloped in most research programs.’

In environmental psychology, the relationship between physical space and the human psyche is the very focus of attention. It has been recognized as an independent subfield since the 1960s (Steg et al., 2013, p. 2). Throughout the history of the discipline, environmental psychologists have sought to understand how physical environment affects the human psyche and especially its well-being (Steg et al., 2013, p. 3). Quite often, the research has had an applied orientation, with the goal being to design offices and other buildings in a manner that facilitates optimal performance and well-being. The context of the workplaces has been a particular focus (see, for example, Clark et al., 2014; Xu et al., 2020; Flatau-Harrison et al., 2021; Kao et al., 2021; Wang et al., 2022; He et al., 2023). Another key concern has been environmental protection. As awareness of pollution and climate crisis has increased, so have psychological attempts at promoting environmentally sustainable attitudes and behaviors.

When psychologists first started to investigate how the physical environment influences behavior, they sought to gather data from human activity in its natural surroundings (Bonnes and Bonaiuto, 2002, 29), and the methods of choice were field experiments and observation. In contrast, the laboratory-based methods commonly utilized in psychology were largely shunned by environmental psychologists. Because of removing participants from their natural environment, laboratory studies were seen as producing results that were neither socially relevant nor ecologically valid (Bonnes and Bonaiuto, 2002, p. 29).

From the beginning, environmental psychology has also been characterized by interdisciplinarity and openness to borrowing approaches from neighboring fields (Bonnes and Bonaiuto, 2002, p. 29; Steg et al., 2013, p. 5). Cultural anthropology, ethnology, sociology, geography, and architecture studies have been among the key discussion partners. Today, environmental psychology is characterized by methodological pluralism (Steg et al., 2013, p. 6). In contrast to many other subfields in psychology, it has no one dominant method, and field studies, questionnaires, experimental designs, and computer simulations are in use.

The so-called transactional approach has provided the main paradigm for environmental psychology since the 1980s. Bonnes and Bonaiuto (2002, p. 30; see also Heft, 2012) summarize its main starting points: (1) The unit of analysis is a person-in-environment. (2) The person and their environment are two parts of a whole and effect a constant transformation on and of each other. (3) Stability and change are continuous and coexisting. (4) The direction of change is not predetermined but emerges from the person-environment interaction. And, (5) a change occurring in one part of the person-environment system reverberates through the whole system.

The transactional approach has been criticized for being too abstract and difficult to apply in actual research. In order to bridge the divide between principle and practice, environmental psychologists are increasingly arguing that the environment should be studied not just as physical but socio-physical (Bonnes and Bonaiuto, 2002, p. 30; Heft, 2012, pp. 19–20; Valera and Vidal, 2016, p. 44). In other words, research should consider the social dimensions of a physical space.

Like the subfield more generally, much of emotion research in environmental psychology is motivated by ecological concerns and the design of buildings that promote human flourishing (Qiu et al., 2020; Chen et al., 2022; Ojala et al., 2022). For example, Bower et al. (2019) have done a systematic review of studies investigating the impact that visual properties of built environments have on the emotional states. Despite the reviewed studies being few in number, and limited in terms of sample size, the authors were able to draw some interesting conclusions; for example, rooms with curved lines and high ceilings evoked more positive emotions than rooms with horizontal lines and low ceilings.

A fruitful way to approach the sense of safety in religious settings is to conceptualize such settings as “places.” In environmental psychology, it is exactly the term “place” that is used to refer to the whole formed by a physical space, its communal uses, and the individual meanings that are associated with it (Bonnes and Bonaiuto, 2002, p. 30; Hunziker et al., 2007). Social and environmental psychologists have investigated placemaking as an important aspect of identity construction and intergroup boundary work (Hopkins and Dixon, 2006, p. 176). Social identities are very often spatialized, in the sense that one’s degree of belonging to a place is determined by membership in a group or social category. For example, the extent to which one enjoys the right to participate in the nation-state depends on one’s citizenship but also on other social identities that intersect with it, in particular, gender, sexuality, class, race/ethnicity, age, and (dis)ability.

Such social identities are thoroughly embodied. Thus, different bodies have different possibilities to belong in any given context. Belonging is indeed crucial in exploring sense of safety. It is a multifaceted concept that includes both practical and emotional aspects (Yuval-Davis, 2006, p. 199). On one hand, belonging is closely related to social and political agency as well as one’s rights in the community. When a person is perceived as belonging, they have a say in the community’s affairs. In addition, they often enjoy protection that others do not have. On the other hand, belonging often feels like being “at home” and evokes emotions such as peace and contentment (Yuval-Davis, 2016, p. 369). Of course, this is not always the case. Besides warmth and joy, “home” may also cause anger, resentment, and anxiety. Religions and religious places and spaces epitomize such duality in a fascinating manner.

For the study of sense of safety in religious spaces, environmental psychology has several methodological implications. First, in order to attain ecologically valid data, human behavior should be investigated in its natural context. Second, the relationship between a person and their environment is determined by physical, psychological, and social factors. Accounting for this complexity requires multidisciplinary and multimethodological approaches. Third, in person-environment interaction, neither physical, psychological, nor social factors are primary; rather, they intertwine and interact. In analyzing them, one should avoid approaches that reduce relationships between variables to simple causal effects. As Lynch (2020) has argued, sense of safety is a gestalt-like system—a comprehensive experience of feeling safe in one’s body, space, social relationships, and mind, without any of these domains being primary.

## 4 Explorations of sense of safety in relation to space: urban studies

Safety in and in relation to urban space is one key aspect of the multidisciplinary field of urban studies, especially of urban planning, and urban sociology. Urban spaces are usually also diverse, which makes them an appealing context for understanding experiences and negotiations of space from the viewpoints of co-existence and its challenges among different groups and identities, including religious ones. In this multidisciplinary field, sense of safety (as well as other emotions) has been researched especially in relation to urban planning, livability, and wellbeing. A significant body of literature investigates the role of environmental features, social environment, and / or social features (e.g., gender, ethnicity) in sense of safety. Sense of safety is in this body of research approached mainly as an individual estimation, perceived safety, or sometimes explicitly related to fear of crime (e.g., Azevedo et al., 2020; Jedon et al., 2022; Lu et al., 2023; Ratnayake, 2017).

Gender, nationality, and ethnicity have been found to influence the experiences of sense of safety in urban space at least in American, European, and Asian contexts. This connection is explained by vulnerability. For example, women, the elderly, and minorities experience vulnerability more often. Similarly, victimization experiences, both direct and indirect, increase feelings of unsafety (e.g., Azevedo et al., 2020). It has also been found that structural factors, such as population density, inequality, neighborhood disadvantage, and lower levels of social capital and civic engagement can impact people’s sense of safety. Also features of the urban context, such as urbanization rate, population density, air quality, and tree cover can impact people’s sense of safety (e.g., Kuo et al., 1998; Collins and Guidry, 2018; Lu et al., 2023).

More specifically, in relation to religion, sense of safety has been researched especially in relation to minority-majority positions. This body of research investigates the location of religion, urban governance, and power relations. On one hand, it focuses on the ways in which religion is shaped and constrained, and on the other, on how religions shape urban space and materialize and enact religious ideologies and communities (e.g., Burchardt et al., 2023a; Kong, 2001). While the focus of these investigations is rarely on emotions *per se*, emotions are a central part of power relations, minority identities, and placemaking which are all central approaches in this body of research (e.g., Burchardt et al., 2023b; Dilger et al., 2020). Sense of safety or the lack thereof is related especially to those who are read as visibly Muslim in Western cities. Listerborn (2015) writes about the continuous potential threat of violence in public space, which is embodied and physical (on geographies of islamophobia see special issue edited by Kawtar and Hopkins, 2019).

The visibility of places of worship or rituals of religious minorities in urban space has been researched as sites or symbols of recognition as well as conflict and contestation in diverse contexts ranging from India to European countries and from China to the Americas (Burchardt et al., 2023a). Thus, social norms and relations materialise in the negotiations, opposition, governing, and allowing of religions in public (urban) space, highlighting also the viewpoint to space as socially produced (Dilger et al., 2020, in African context). Recognition and belonging are negotiated processes in which cohabiting and co-building places are central. These places are often religious, especially in the case of diaspora communities and people who have migrated to a new country and are thus ‘out of place’ as Vasquez and Knott (2014) formulate it in their research covering urban contexts in Malaysia, UK, and South Africa. Religious buildings become spaces for the construction of identity (Vásquez and Knott, 2014). The resistance to allowing (minority) religious buildings, clothing, or the use of public space for religious processions or festivals represents exclusion of (certain) religious identities, affecting the sense of safety of those representing them (e.g., Bradamat et al., 2022; Burchardt and Becci, 2013; Burchardt and Westendorp, 2018). Religions can also be racialized adding to the ways in which a minority position intertwines with threats to sense of safety.

For the study of sense of safety in religious spaces, we can draw several implications from urban studies. Similarly as pointed out in the last section, urban studies show how sense of safety intertwines with physical, psychological, social, and societal factors, and often with power relations. To understand the experiences of sense of safety in religious spaces, individuals’ experiences, positions in different communities and in the society, as well as the ways in which these play out in and intertwine with the materiality of their context, need to be studied. To develop a model for investigating sense of safety in religious spaces, individual experiences and their social, societal, and material contexts need to be investigated with multidimensional tools.

## 5 Recognition and politics of belonging to enrich the methodology of spatial investigating sense of safety and religion

For the reasons explicated in our Introduction, this segment of the article draws from a more theoretical angle of political philosophy articulated in the contemporary recognition theory and the politics of belonging. The aim is threefold: (1) to initiate a discussion on the recognition of religion in contemporary societies, in public spaces in particular; (2) to examine the interconnectedness between emotions, acts of recognition and religion; and (3) to complement the discussion with the politics of belonging by paying attention to ethical and political standards, which are utilized to assess belonging. As we will argue, the theory of recognition and the politics of belonging are valuable frameworks for exploring the relationship between religion and spatial dynamics, revealing how acts of recognition or their absence can either foster or undermine a sense of safety and inclusion in religious contexts.

It is widely accepted that recognition is a basic phenomenon, having a profound effect on psychology, sociology, politics, history, and religion. In contemporary recognition theory, recognition is understood as a mutual granting of positive statuses between individual human persons. In an extended sense, recognition applies also to groups and institutions (Kahlos et al., 2019). Proponents of the theory typically highlight the idea of the social formation of the self, the importance of positive self-relations, and the authenticity of the self. Philosopher Axel Honneth argues that recognition has three distinct dimensions, all of which focus on particular aspects of personhood. First, regarding respect, recognition acknowledges our identity as rational, independent beings, and is built on the belief of equal dignity. Second, regarding esteem, recognition is based on our identities as individuals of a certain kind with personal, cultural, ethnic, or religious backgrounds, driven by the evaluation of the unequal benefits or merits of these identities. Then, regarding love and friendship, recognition emphasizes the distinctive individual persona of the other; it is founded on unequal personal importance among a smaller group of people with strong emotional ties. These three dimensions have a formative effect on our self-relations, which in turn profoundly affect our capacities to operate as fully functional adult human beings in our societies (Honneth, 1995), also promoting the sense of safety among fellow human beings.

Contemporary recognition theory allows us to analyse the emotions related to the three dimensions (respect, esteem, and close relationships), as well as different forms of misrecognition within all these dimensions and respective reactions to them. Mostly, we are concerned about recognition relations and sense of safety in religious communities. While recognition has been considered as a vital human need, and inherently good, recognition is also comprehended to encompass struggles and debates of uneven or difficult power relations. Such struggles derive from a shared feeling that neither individuals nor the group have had their uniqueness noticed or respected and that within existing ways of acknowledgement, there is not enough room to recognize certain aspects of our being (Honneth, 2002, p. 504). Our research is keen to analyse how these struggles for recognition manifest in religious contexts, especially in religious places. For, example, how are different individuals or groups recognized (respect, esteem) in a religious space through architecture, visual elements, or communication.

Typical affective, emotional reactions rising from recognition are then pride, joy, love and belonging, whereas misrecognition and lack of recognition result in shame, envy, feelings of exclusion and hatred. Thus, arguably, recognition entails several emotional responses which may either promote or erode our sense of safety. Interestingly, these intimate, strongly reciprocal emotions, reveal in their distinct ways the dynamic interplay between private and public spheres, the inner-built reciprocity of emotions and the problematics of identity constitution within individuals and groups. Enhancement of sense of safety, and the erosion of it, may be deeply intertwined—not either or. Perhaps the phenomenon is typical in religious communities, due to the powerful emotional standpoints of many individuals and leaders.

Honneth has suggested that for social justice to be achieved, individuals need to have an atmosphere where they can “appear public without shame” (Honneth, 2004, p. 355, quoting Adam Smith). Sense of safety is thus not only about the absence of perceived threat but also about feeling free to express oneself (see also Lynch, 2020; Lynch et al., 2025). Taking Simon Thompson’s recent alterations to the theory of recognition into account, we posit in this development of novel methodology, that locality is an essential ingredient of any exhaustive description of recognition, including the recognition of religion.

Though much consideration has been given to the giver of recognition, its recipient, its form, and its outcomes, few have investigated the location where it is bestowed. Thompson usefully suggests that the public space is a type of a public good—or, more precisely, that it should be regarded and treated as if it were such a good (Thompson, 2019). He refers to Waldron (1991) who emphasizes: “Everything that is done has to be done somewhere.—Since we are embodied beings, we always have a location.” Spaces and our embodied existence should never be ignored in academic analysis.

Our development of methodology in this article indeed argues that location is essential when one looks after another’s welfare and sense of safety, provides them with jurisdiction over common standards, or values their contribution to common objectives, all of which are required when assessing acts of recognition. The empirical examples and analyses in this article of ours affirm Thompson’s idea of space of recognition being relevant: the person offering it, the way in which they do it, and the person receiving it. The closer look at the space, place, and location of recognition helps us to understand and analyse different affective, emotional responses towards, for instance, changes in public space and the ways in which religious individuals and groups are represented within communal life.

Furthermore, our approach goes beyond just utilizing contemporary recognition theory; we also aim to complement it with the politics of recognition, which specifically focuses on the emotions and sense of belonging, including the sense of safety. While politics of belonging typically refers to citizenship, entitlement and status related to governing, we take two steps forward: one concerning the everyday and one towards a more philosophical perspective. We then investigate the everyday politics of belonging—lived, embodied, spatial, and continuously constructed in the everyday—but maintain the systematic approach deriving from the insights of the contemporary recognition theory. As Yuval-Davis (2006) notes, the politics of belonging is not only about political projects but entails the struggles around the determination of what is involved in belonging, in being a member of a community, and of what roles social spaces and narratives of identity play in this. We firmly believe that constructions of sense of safety in religious spaces make these dimensions of politics of belonging tangible, as emotions are lived in relation to, and in, communities and the wider society.

The lived politics of belonging provide insight into religion’s complex roles in shaping belonging, identity, emotional attachment, and feelings of safety within various communities and societies. This concept also highlights the importance of solidarity, seen in a mutual, emotion-based sense of support and an appreciation of shared interests, goals, standards, and sympathies.

The politics of belonging typically comprises also specific political projects aimed at ‘constructing belonging’ to collectives. According to Yuval-Davis, there are three interconnected levels highly relevant to recognition when considering the creation and experience of belonging: social locations, identifications and emotional ties to various groups and collectives, and ethical and political standards which people utilize to assess their own and others’ belonging. We then underline the fact that the politics of belonging is also about the struggle around the determination of what is involved in belonging, what counts in being recognised as a member of a community and what part social location and specific narratives of identity play in this (McLaughlin et al., 2011; Yuval-Davis, 2006).

Bart van Leeuwen has similarly expanded Honneth’s recognition theory into a trajectory which asks how it feels to be accepted somewhere, thus, emphasizing social attachments. Acceptance is marked by the absence of anxiety and the presence of comfort in social settings, as opposed to the fear of being refused. Such sense of belonging, or being socially accepted, implies sense of safety built on the recognition: that one is connected to a larger group. The formal conception of the good life should take into consideration, on the one hand, the need for autonomy, and the other, the sense of being accepted by the communities in which one lives. Belonging, although often taken for granted by those in privileged positions, is experienced as fundamental to well-being for most people (van Leeuwen, 2007, pp. 195, 199)—a true source of sense of safety.

## 6 Developing a novel methodological approach for investigating sense of safety in religious spaces

As this article has illustrated, negotiations of who belongs and who does not take place on all levels of society, from interpersonal encounters to institutional discourses. With belonging comes sense of safety, both in the concrete sense of having recognized rights in the community and in emotional sense of, for instance, “feeling at home,” and various forms of experiencing sense of safety. Drawing from the above discussed viewpoints, this is visible in how sense of safety is influenced by both personal and societal positions, vulnerabilities, and inequalities.

To conclude from the three exemplary fields above; First, the experiences of urban sense of safety increase with social connectedness. Second, similarly in environmental psychology social identities have been found to be often spatialized, that one’s degree of belonging to a place is determined by membership in a group or social category. The extent to which one enjoys the right to participate in a specific community or group often depends on social identities, in particular, gender, sexuality, class, race/ethnicity, age, and (dis)ability highlighting the embodied nature of social identities. Third, this interplay between sense of safety and right to belong in spaces can be understood in a more nuanced manner with the concepts of recognition and politics of belonging. We can further understand the aspects of belonging through investigating how individuals experience recognition: respect, esteem, and close relations within a religious space, and through investigating how communities or groups—which materialize societally and publicly as religious spaces—are recognized societally. Politics of belonging complements this approach of recognition theory with the specific viewpoint of ethical and political standards, which are utilized to assess belonging. The research from both fields of urban studies and environmental psychology also highlight the embodied interplay between emotions and (physical) space. The material, embodied features of space, in addition to its social aspects, influence sense of safety, and in some part intertwine with the social aspects.

Based on this, to empirically investigate sense of safety in religious spaces, we propose the following spiral approach, applicable for both data collection and analysis. It is a methodological tool built especially on the perspectives of spatial methodologies as an analytic viewpoint, environmental psychology, urban studies and the concepts of recognition and politics of belonging applied to our subject matter. To its core lies our understanding of all emotions as social, societal, and spatial. Our novel *one-plus-five dimensions tool for empirical explorations of sense of safety in religious spaces* is, as follows:

(1) Identifying a *religious place*. A place refers to a specific space or location with a meaning, which is important for a person or a community, and often to identity construction as social identities often spatialize in specific places. Thus, “a place” is not just any space or material setting, but one that has special relevance and meaning to a person and/or a community. In this stage, research participants can be asked to identify places that they consider to be sacred or otherwise of marked religious/spiritual/existential significance to them. On the other hand, research participants can also be asked to evaluate the significance of a specific religious space to them. Interviewing, questionnaires, and participant observation are all potential methods for data collection.
(2) Social ties and belonging play a fundamental part in sense of safety; Thus, *intergroup connectedness*. Unpacking intergroup connectedness, and networks of belonging and safety, as well as boundary construction and how they are afforded by physical surroundings. Social identity construction usually includes intergroup boundary work, and from the viewpoint of placemaking, this intergroup boundary work can correspond with the spatial boundaries of the place. Belonging to a place is thus determined by membership in a group or social category and their recognition, which usually intertwine with the theologies or teachings of the community. These categories often intersect with other social identities such as gender, sexuality, class, race/ethnicity. On the other hand, there can be different levels of collaboration and connectedness with other groups, which can also relate to the physical surroundings by, for example, access to the place. The investigation of intergroup boundary construction and its relationship with the religious place focuses on identifying the spatial boundaries of the religious place and how these spatial boundaries correspond with intergroup boundaries. The researchers can investigate how the intergroup connectedness, intergroup boundaries and their relations to the spatial attributes and boundaries are negotiated in the community or people using the space: Who is perceived as belonging to the community and the place? What kind of rights and responsibilities are associated with belonging? Who can access the place and participate without belonging to the community? Particular attention needs to be paid to the ways in which physical surroundings signify or contradict an intergroup boundary. For example, what languages are used in signs that are displayed in the space? Also, the relationship of the community with the surrounding societal context is investigated, for example, in relation to inequalities and majority-minority -positions as well as collaboration. All are crucial questions in order to understand, or to promote, sense of safety. The primary method may be participant observation, which can be supplemented with interviewing, document analysis, etc.
(3) Also, focusing on *intragroup boundaries*, and how they are afforded by physical surroundings, is a needed step in order to dive into the intricacies of sense of safety. Quite often, there is no clear-cut divide between belonging and not belonging. Instead, there are several degrees of belonging, with some people (e.g., white middle-aged heterosexual men) enjoying a more privileged position in the community than others. Individuals also negotiate their level and dimensions of belonging in relation to the community, and this can spatialize in the ways in which the space is used and belonging experienced. In this stage, the analysis focuses on intragroup hierarchies and how they are constructed both discursively and spatially. For example, are there different degrees, levels, or types of belonging? Is there equal respect for all members of the community and how is esteem distributed within the community? Does the level of belonging vary and why? To what extent is diversity in the community recognized? Are there some identities, the legitimacy of which is either implicitly or explicitly denied? Are the needs of all community members taken into account in the planning of the religious space (e.g., are there differences between the spaces designated for men and women, respectively). And how does this intertwine with the teachings and understandings of the community? Methods then can include participant observation, interviews, surveys, and document analysis.
(4) Exploring the *embodied emotions* that are associated with the place and its spatial dimensions. The focus then shifts to emotions and, in particular in this case, the sense of safety. The viewpoint of emotions binds and concludes the previous stages together: How do the material characteristics of a space, the people who occupy it, and the different representations and (intra- and intergroup) discourses associated with it affect or intertwine with sense of safety when investigated through embodied emotions in the space? Does the religious space feel safe to them? Or the contrary? Special attention is thus given to the experiential and embodied aspects of emotion, which intertwine with personal histories and experiences of safety and unsafely (e.g., victimization or violation of integrity, Azevedo et al., 2020; Lynch, 2020)—especially in relation to the place and its spatial dimensions including its social and societal dimensions. And, how does it actually *feel* to feel safe? In cognitive terms, sense of safety is a complex emotion, as we noted above. Thus, sense of safety has certain biologically hardwired “core” that provides it with a phenomenological tone. However, what ultimately distinguishes sense of safety as a distinct emotion are culturally coded meanings and interpretations that are associated with it. The goal of the analysis at this stage is to unpack sense of safety into its constituent, cultural, embodied components: What kinds of thoughts, beliefs, feelings, and so forth, make up the lived experience of safety for each research participant? These include traces of earlier times, different stratifications, and questions of power and struggles related to the spaces investigated, that can be investigated and analyzed using the concepts of recognition and politics of belonging. Interviewing is a key method of data collection, but it can be beneficial to supplement it, for example, with photovoice and other creative participatory approaches which enable versatility to the means in which participants can share their embodied emotions. Emotions indeed are not often easy to verbalize.
(5) Looking at the embodied emotions of sense of safety of inter- and intragroup nexuses *in the framework of wider social, societal, and global vistas.* As illustrated in our opening: All emotions are experienced individually but always also influenced by social factors as well as by wider cultural, societal, and political frameworks. Similarly, societal and political debates concerning sexual rights play a role in individuals´ fears and experiences of sense of safety. Such issues might not always lend themselves to being articulated as specific interview themes or questions, nor survey items, but researchers must grasp their significance. And, we have frequently encountered instances where socio-political contexts, including global ones, have become focal points during interviews, initiated by our interviewees.

Could this be a model for exploring any organizations and communities? Basically, believe this methodological tool can be applicable to also other contexts than religious ones. However, as our interest lies specifically in investigating sense of safety in religious spaces, the spiritual, transcendence-related dimension in central in all five dimensions. For instance: What is an individual’s experience of their spirituality and spiritual sense of safety? How do one’s religiosity, spirituality, and spiritual sense of safety (or lack thereof) affect and intertwine with and throughout the dimensions? Having a strong sense of safety in one’s experienced relationship with the transcendence can, for example, mitigate or contradict experiences of unsafety resulting from intergroup, intragroup or societal dimensions of sense of safety in a religious place. Thus, the viewpoint of individual and/or communal transcendence and spirituality penetrates all dimensions and should be taken into account when collecting data on them. In the best case scenario, individuals may experience (what could be called) spiritual sense of safety in their communities. In our model we have named this `deeper´, more existential, religion- and spirituality-related, penetrating level `transcendence´. The concept refers to both individuals and communities better than, for instance, the concept of `religiosity´ or `spirituality´. And, thanks to this one over-arching, penetrating dimension, the name of our model: *a one-plus-five dimensions model.*

**Figure 1:**
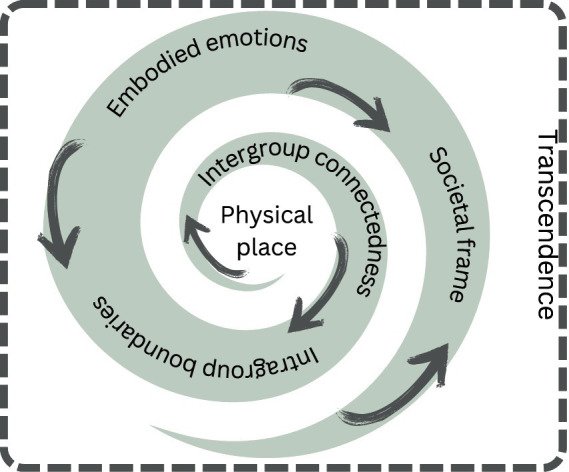
One plus five-dimensions spiral model for empirical explorations of sense of safety in religious spaces.

We believe there is certainly linearity in these one plus five dimensions; For instance, the fifth one serves both as a starting and an ending point of all explorations—yet, it may not be a separate issue in the interview guide. Furthermore, some interviewees might find it easier to approach their experience deductively (moving from wider issues to smaller ones), while others do the opposite, inductively. Additionally, particularly the 2nd and 3rd dimensions may be deeply intertangled. In essence, the dimensions ought not to be viewed or utilized in isolation, nor should they be strictly adhered to in a sequential manner, as depicted in our 1 + 5 spiral illustration (Figure 1).

Such a methodological tool aims to bring together the physical/embodied, social, societal, and mental dimensions of both emotions and space, with the intention of informing us on how sense of safety is constructed and contested in—and in relation to—religious spaces.

## 7 How to apply the spiral model on concrete case examples of sense of safety

We will in the following introduce four empirical case studies to demonstrate how our spiral model may be applied; We wish to share a novel tool, not just the end results. The four case studies are all ongoing, and they are (1) a novel communal church building, (2) LGBTQ+ muslims, (3) Jewish mikveh baths, and (4) intersections of religion and dance works. Together these case studies hopefully illustrate the versatile opportunities of the above introduced model. In the future, these four cases will produce their own findings; No comparative material is required in order to use our spiral model.

### 7.1 Example case 1: Communal church building and sense of safety

The Tikkurila Church is a new church building and a sacred space, completed in 2021. The church represents Lutheranism, the majority religion of Finland, and is located in one of the most multicultural neighborhoods in the capital region. The church is situated prominently in the urban space, along a pedestrian street, directly across from the market square and the city hall. In the same quarter, owned by the parish, student apartments were built simultaneously, with the aim of creating a communal, very centrally-located city block. The church’s lower lobby features a trendy café and an open lounge for families with children. Its architects state: “The interior was designed to be relaxed and inviting, with the aim of avoiding solutions that would make the church look like a municipal building.”^1^ Entering through the main door, people encounter a bright, open space where they can linger, read newspapers, sit on a sofa, move to the church hall for contemplation, or sit in the café.^2^

The design of the Tikkurila Church prioritizes creating an inviting space intended for visitors, regardless of their affiliation with the congregation. Proximity, openness, and inviting atmosphere have been the fundamental principles of its architectural design (Sun, 2019). The concept design of the church was carried out using service design methods,^3^ with a focus on user-centered consumer perspectives. Citizens were asked about the type of church hoped for or needed: What they wanted to see inside, and what functions the church should offer.^4^

In the case of the Tikkurila Church, we will explore the viewpoints of the users with a qualitative, open-ended survey, and interviews. They will both explore the religious / spiritual significance of the space for the informants (applying the step 1 of the model). Do the users ascribe spiritual, religious or otherwise existential meanings to the building? Does this vary between the actual church space and the communal space? How is the space used, and what kind of meanings does it gain in the city space? We also include the viewpoints of (2) inter- and (3) intragroup boundaries and their relationship with the space in the survey and interviews, complemented with participant observation, as we investigate the informants’ negotiations related to different religious backgrounds (e.g., active congregation members and occasional visitors), and groups of (possible) users of the space. Who is viewed to belong to the space, who is not, are differences in the levels of access, position, or belonging to the space, and does this vary in the different parts of the building (religious / communal) or at different times? Moving on to stage (4), we will ask the informants about their emotions and experiences associated with the space, particularly sense of safety, and the relationship between these emotions and the space. The interviews will be done in the church space to invoke experiences which bridge the emotions with the space.

### 7.2 Example case 2: LGBTIQ+ Muslims, sense of safety, and religion

A number of studies have demonstrated that Islamophobia and anti-Muslim racism are very widespread in Europe (see, for example, European Union Agency for Fundamental Rights, 2017; Pew Research Center, 2018). For example, in Finland, over half of the population report that they have at least somewhat negative attitude towards Islam (Pauha and Ritola, 2022, p. 198) and almost two-thirds perceive Islam to be fundamentally incompatible with Finnish culture and values (Pew Research Center, 2018, 21). For many European Muslims, mosque provides a safe space in the middle of a society that is suspicious of Islam. However, mosques are not safe spaces for everyone. Intolerance towards sexual and gender minorities is common in diaspora communities from Muslim-majority countries (Van der Bracht and Van de Putte, 2014, p. 118; Röder, 2015, p. 1062; Soehl, 2017, p. 1832). Importantly, mosque attendance is a strong predictor of negative attitudes towards homosexuals (Röder and Spierings, 2022). Those who are most active in the mosque are on average also the most hostile towards sexual minorities.

Despite the prevalence of homo- and transphobia, however, LGBTIQ+ Muslims have found, and often actively established, spaces in which to feel welcome and safe. The internet, and social media in particular, have become important sources of support and community for many of them, as they have for geographically dispersed and marginalized minorities more generally (Linjakumpu, 2011, p. 44). While still few, there are also mosques that are explicitly inclusive of sexual and gender diversity. Furthermore, spaces can be religiously or spiritually meaningful despite not being ‘religious spaces’ as the term is commonly understood. For example, for many of the LGBTIQ+ Muslim participants in Yip and Khalid’s (2010, p. 167) study, their own home was such a space. The same study also showed that sometimes a spiritual space has no fixed physical location or boundaries. Instead, a spiritual space can simply be one in which a Muslim is in encounter with their God.

In our project, we investigate the experiences that LGBTIQ+ Muslims have regarding safety (or lack thereof) in religious spaces. Following our spiral model, we commence our investigation by mapping out the variety of spaces that hold religious, spiritual, or otherwise special significance to our informants (Stage 1). We will pay special attention to how our informants negotiate their belonging in various communities and the associated spaces (Stage 2). In this initial mapping, we utilize primarily online questionnaires and semi-structured interviews. We begin by asking our respondents to think of places that hold a religious, spiritual, existential, or otherwise special meaning to them. After gaining a description of these places and the activities conducted therein, we invite our respondents to reflect on questions of ownership and belonging: Who are the other people coming to the place? Who owns it?

With regard to gender and sexual minorities, many mosques adhere to a” do not ask, do not tell” policy; LGBTIQ+ Muslims are able to participate in mosque activities as long as they keep their sexual or gender identity to themselves (Thompson, 2020, p. 124). Even in the absence of acute threat, however, not feeling free to express one’s authentic self undermines the sense of safety (Lynch, 2020). Moreover, hiding one’s sexual or gender identity may not be an option for everyone. For example, spatially, mosques typically have separate sections for men and women, and transgender Muslims may feel excluded in both spaces (Kumpasoğlu et al., 2022, p. 10). The example of transgender Muslims illustrates well how certain kinds of bodies are excluded from belonging in a religious space and how sense of safety is therefore embodied. Assessing the degree of belonging that different kinds of bodies enjoy in a space (Stage 3) is an important part of our investigation. We address this issue through interviews but also by conducting participant observation in a variety of Muslim religious spaces. The questions we ask concern, for example, the explicit and implicit norms for acceptable behavior in the place, as well as the needs of different kinds of people and how these are met in the design of the place.

Together, stages 1–3 lay the foundation for analyzing the embodied emotions associated with various spaces (Stage 4). For an LGBTIQ+ Muslim, worshiping at a mosque may compromise one’s sense of safety in more than one way: On one hand, being open about one’s sexuality or gender identity may subject them to psychological, spiritual, or even physical violence. On the other hand, the pressure to conceal important aspects of one’s identity is burdensome and disquieting. In the absence of physical mosques that are inclusive, virtual communities provide an important religious setting for many LGBTIQ+ Muslims (Linjakumpu, 2011, p. 44). However, to study embodied emotions in a virtual space poses a significant challenge. How to conceptualize embodiment in a setting that is, by definition, disembodied?

It is worth noting that while LGBTIQ+ identity is, for many, a source of religious discrimination, it may also be a source of religious growth, empowerment, and self-transcendence (Yip and Khalid, 2010, p. 157). Working through the tensions between one’s learned tradition and personal gender/sexuality may help one grow into a confident and outward-looking believer for whom being loved by God is a felt reality. In this way, marginal sexuality or gender may be experienced as a gift from God (Yip and Khalid, 2010, p. 152).We tap into the embodied experiences of our informants by interviewing them. We ask about emotions that the respondents have felt while in the place or that are evoked when they think about it. We also ask them to describe the emotions, their nuances, and situational variability. In addition, participant observation and creative products, such as poetry and art, may provide insight into those experiences that resist being put into words.

Finally, in addition to grappling with homophobia and transphobia in the Muslim community, LGBTIQ+ Muslims in Europe also need to cope with discrimination and prejudice in the broader society. Besides sexuality and gender, such prejudice may also be based on the ethnicity or religion of our informants. In Stage 5, we analyze how general societal dynamics, such as the rise of right-wing nationalism across Europe, affect the sense of safety of LGBTIQ+ Muslims in religious spaces. If the interviewees have not spontaneously discussed their feelings of belonging and safety in the broader society, we ask about them at the end of the interview.

### 7.3 Example case 3: Sense of safety in the waters of the Jewish mikveh

In researching contemporary Jewry, the question of safety is widely analysed in relation to issues of antisemitism and hate crimes towards Jews (Dencik and Marosi, 2017; Shainkman, 2018). To complement this statistic information on safety with an in-depth understanding of how a *sense of safety* is created, negotiated, and upheld in Jewish life today, ethnographic investigations provide important elucidations. In this case study, the spiral model for methodologically exploring the sense of safety within religious spaces will be implemented to highlight a Jewish space of sensitive salience: the *mikveh*. The ritual bath was long rejected by non-Orthodox Jews as an outmoded ritual based on obsolete purity laws and exhaustingly complicated rules upholding rigid, non-inclusive apprehensions of gender and sexuality. Today, however, ritual immersion is spurring novel interest among liberal and progressive Jews, a tradition to be rediscovered and re-evaluated for its potential to create deeply emotional and embodied spiritual experiences (Dorff, 1999; Baskin et al., 2007; Martin, 2022).

To clarify why, when and how Jewish women in the Nordic countries choose to visit the mikveh, the first phase of the analysis will explore the mikveh as a “place” in the deepened sense of the word outlined above, together with the research participants. Secondly, intergroup boundaries relevant to this space will be approached, posing the questions: Can Nordic Jews, who neither subscribe to the Jewish law (*halakha*) in an orthodox, compelling sense nor locate themselves with ease on the traditional gender binary, find ways of being in this religiously significant space? Can they perform the age-old rituals in ways that feel genuine and meaningfully rooted in tradition, but still relevant in their individual lives, inter-personal relations and communities?

In the third phase, boundaries within the group come to the fore, connecting the physical space of the mikveh with in the inter-personal discursive space created around it and to recognition. Does the mikveh endow its visitors with a sense of safety regardless of whether immersion is sought for traditional reasons (ritual cleansing related to the menstrual cycle) or contemporary, personalized ones (e.g., after a divorce or retirement)? Are all ritual needs equally recognised as warranted and sincere by the institutions upholding the baths? The fourth step will uncover the inner, emotional meaning-bearing spaces formed by the individuals in relation to the mikveh and the embodied experience of being in this space. The assumption is that the mikveh can serve as an emotional bridge to the past, to generations that have gone before and to Jews all over the world, which brings in the fifth and final step of the analytical model (traces of earlier worlds as a dimension of space as conceptualized by Knott, 2014). Thus, it combines the personal search for a spiritually meaningful practice with the wish to rejuvenate communal resources, nurtured by an emotional investment in a distinctly embodied existential practice (Polak-Sahm, 2009; Muir et al., 2023). In all stages, interviews, participant observation and field diaries can be used as methods for data collection.

### 7.4 Example case 4: Dance, religion, and sense of safety

Dance is a form of art that is exceptionally capable of transcending boundaries between the known and unknown, between the body and embodied experience, and between oneself and another (e.g., Kieft, 2014). This transcending capability of dance has also been consciously utilized to transcend boundaries between worldviews and religions (Mercadante, 2017). Dance entails political power, to bring together people, lands and creatures without erasing their unique qualities, and to enhance the sense of solidarity (van Mourik Broekman et al., 2019; Hellsten, 2022). Dance as an embodied practice can be a way of being religious (Gaston and Gaston, 2014) and a spiritual practice (Gronek et al., 2023).

For dance works, there are some audience-reliant properties or features. Philosophically, it can be discussed whether audience is a central or a defining feature of a work of art, in the first place (McFee, 2019). The focus on the audience experience—like in our forthcoming research—offers a viewpoint to not only the individual experience but the collective embodied space, and even the claims, artistic and religious, the dance work creates in its own right (see Lepeigneux, 2022, pp. 97–109). There is a significant gap in the research of audience experiences related to religious contexts and the sacred, as well as sense of safety and the spiritual in dance artworks in more general.

Research approaches to audience experience often combine kinesthetic empathy, experiences and emotions during a performance, and speaking about these experiences (e.g., Artpradid, 2023). The experience can be both physical and artistic—or be formed by an aesthetic experience and kinesthetic response (Vukadinović and Marković, 2022). This can entail movement but also non-movement: collective stillness momentarily sensed by everyone in the room. This has been observed to be a key signal of audience engagement (Theodorou et al., 2019). To explore the sense of safety, a comprehensive approach should also take into account audience emotion, which is a complex social phenomenon. It has been described as collective, active, dynamic, reflexive, spatial and temporal as well as shared and contingent (Kolesch and Knoblauch, 2019).

In our approach, we will collect material from the audiences of two works of dance: one in a traditional sacred place (a church building from 19^th^ century) and with a theme rooted in the transcendent aspects of life, and the other with the theme of faith, a flamenco dance work exploring the texts of 13th-century poet and mystic Rumi. With, for instance, a discussive approach, a group interview with the members of the audience as well as the artists right after the performances, we will examine, (1) how the members of the audience construct the space around them in terms of spirituality and sense of safety. For example, we will organize two dance works in distinctly religious and a non-religious place, and invite the audience members in the survey to explicate, how the place affected their experience. Here our focus lies in investigating the extent and focal spatial elements of religious meaningfulness of religious place. Then we move our focus to both (2) the inter-group and (3) intra-group boundaries and togetherness the informants experience. For example, we ask respondents to provide information on how frequent dance event goers they are, and to elaborate on their experiences of familiarity and unfamiliarity in their participation. Here we are able to dig deeper into, what creates a sense of belonging and mutual recognition. And lastly, we will (4) map the informants’ emotions related to the experience, to uncover the emotional space linked with sense of safety. This will be carried out by asking about emotions felt directly on the survey on both of the dance works, but also discussing experiences and emotions in the group interview event after the performance.

## 8 Discussion

With our development of this methodological approach, we wish to advance a more holistic view of humans in research. Rationality of humans over emotions still over-dominates in academia, both explicitly and implicitly. Yet, emotions are both an inevitable, fundamental universal human feature and a social-constructively learned set of experiences (Plamper, 2015). Taking emotions more seriously has true scientific potential, particularly in the study of the sense of safety. The sense of safety is not (only) based on rational risk or threat calculations, but it is a comprehensive experience of being safe in one’s body, surroundings, social relationships, mind, and the universe at large (Lynch, 2020). Thus, the motivation for this article has risen from pondering: How to better study empirically the ways in which sense of safety is being constructed and contested in religious spaces.

As discussed in the introduction, any space is the sum of its material characteristics, the people who live in it and move through it, and the different representations and discourses associated with it (Knott, 2014). Spaces draw together physical, social, and mental dimensions, which we can analyze as we investigate specific places. Spaces also include traces of earlier times, different stratifications, and questions of power, struggles in which groups or individuals seek to express themselves. Often, one’s experience of a place depends on one’s intersectional positioning; for example, studies demonstrate that mosque design typically serves primarily the interests of men, and the spaces for women are too often left wanting in terms of aesthetics, functionality, or both (Mohammed, 2024). Moreover, as one’s identity can be complex and conflicted, so too can one’s relationship with the space: for instance, for an LGBTIQ+ Muslim of immigrant background, a mosque can be, on the one hand, ‘a home away from home’ and a space in which to meet people who share one’s language and traditions, and, on the other hand, a setting in which one needs to put on a hetero- or cis-normative facade for fear of being harassed.

Our cases exemplify contested constructions of sense of safety as all of them are about minority identities and vulnerabilities at different levels of communities and society. They include various intersections of minority ethnic, religious, as well as gender and sexual identities. The emotional sensitivity and provocative nature of our four cases motivate our take on constructions of sense of safety as ‘contested’. We challenge the typical dichotomies related to religion and sense of safety; We have, and will, scrutinize religious spaces as prisms of simultaneous belonging and exclusion, cohesion and polarization, togetherness, and insecurity. Sense of safety captures all these shades.

To sum our results from the cross-discipline investigation, we propose a one-plus-five dimensions spiral model to empirically explore the dimension of space, particularly in its relation with the sense of safety within religious spaces. The five dimensions are as follow:

(1) Investigating the religious or spiritual meaningfulness of *spaces*: identifying physical places, which are experienced as religiously meaningful or investigating the extent of religious meaningfulness of religious places (= theoretical viewpoint of personal identity in space).
(2) and 3. investigating the places in relation to, and as, *social space* (empirical and theoretical viewpoints of social identity through inter- and intragroup negotiations, recognition, and politics of belonging = discourses and representations).
(3) investigating the experienced, *embodied emotions* related to social space (= experiences of belonging/not belonging as safety/unsafety, mental dimensions of space and belonging).
(4) Looking at the embodied emotions of inter- and intragroup nexuses in *wider social, societal, and political* vistas.

And, our model’s `plus one´: In addition, and very importantly, as the focus of the model is to investigate religious spaces, the viewpoint of *transcendence* (referring to both individual spirituality as well as the religious nature and identity of the communities to be explored), including spiritual sense of safety, needs to be taken into account in all dimensions of the model.

Importantly, all in all, the model’s *spirality*: the dimensions are neither separate nor moving just from 1.-5 but also 5.-1. The fifth one serves both as a starting and an ending point of all explorations, and not as a separate framework.

The spiral model enhances comprehension of social space through philosophical conceptualization and analysis related to the dynamics of recognition and the politics of belonging. Similarly, all dimensions of our model also aim at identifying the interplay between social and material space through material and embodied approaches. We will need to understand it thoroughly in order to understand—and even more so if wishing to promote—sense of safety in religious communities.

Our approach is multi-disciplinary and ambitious. It may be utilized both in data collection and analysis, we hope. We combine individual, communal, and societal viewpoints, and their synergy with material and philosophical approaches, to understand experiences of sense of safety. We firmly believe that all these steps need to be included to empirically understand the different levels of sense of safety in religious spaces, and will test this with our on-going cases. Yet, we additionally suggest these analytical steps can also be used selectively in different research approaches. For example, empirical investigation can focus specifically on the viewpoint of sense of safety as belonging (our step 2) and how it manifests in relation to physical space. In any case, we highlight that also the other steps, viewpoints should be acknowledged when researching the multifaceted phenomenon of sense of safety in religious spaces, even when they are not the primary focus of the investigation.

The approach developed in this article is far from complete, and it is indeed under development in our current empirical-philosophical work presented above. This article is our first analytical attempt to formulate a methodological approach for investigating the multi-dimensional and complex subject matter empirically. As the fields we are drawing from are broad and diverse, and as we aim at combining on a deeper than usual level the empirics and philosophy, we have not addressed them comprehensively. Also, while we have utilized research literature from different religions and different parts of the world in developing the model, our case studies represent certain religions and contexts in the global north. The applicability of the model in researching different religions and contexts should be further discussed.

Various questions follow; Could our spiral model be utilized also in studies of different emotions in the (always) complex, versatile contexts of religion? Most likely this would be the case, at least in studies on various emotions related to belonging and recognition. Also, might our model be a fruitful academic tool in studies of emotions (of at least sense of safety) in other context than religion and religious institutions. For this our firm belief is: Yes.

Additionally, the fundamental question related to sense of safety and religion in space pertains to the rights of religious communities and individuals to express their religion in public space and how such activities should be regulated. Should at least some of them be positively recognized, so that measures can be taken to make sure that believers can practice their religion? More emphatically, should their faith enjoy positive avowal in the public arena and to which extent should individuals be able to express their religious identities in public spaces of their societies. We suggest our model could be applied also in practical endeavors to promote sense of safety.

As many possible and interesting fields and viewpoints of inspiration had to be left out, the work continues. Various streams and approaches would and will be inspirational. Empirical exploration of sense of safety is currently paramount and pressing—socially, societally, and globally. We encourage scholars to test, criticize, and further develop the model we have suggested above—also together with us.

## Data Availability

The original contributions presented in the study are included in the article/supplementary material, further inquiries can be directed to the corresponding author.

## Footnotes

^1^Tikkurila Church is a meeting place open to everyone | Design Stories (finnishdesignshop.com).

^2^Yksivuotias Tikkurilan kirkko on ylittänyt monien odotukset – kirkosta on tullut matalan kynnyksen kohtaamispaikka eri-ikäisille vantaalaisille - Kirkko ja kaupunki.

^3^Ajatus Vantaan srkyn viestinnän kehittämiseksi nykyisillä resurssiella (evl.fi).

^4^Tikkurilan kirkkokortteliin haetaan ideoita palvelumuotoilulla - Kirkko ja kaupunki.
